# Assessing a Public Health Intervention for Children in Barbados, 2003–2008

**DOI:** 10.5888/pcd12.150120

**Published:** 2015-08-27

**Authors:** Jennifer H. Bushelle-Edghill, Sarah B. Laditka, James N. Laditka, Larissa R. Brunner Huber

**Affiliations:** Author Affiliations: Sarah B. Laditka, James N. Laditka, Larissa R. Brunner Huber, Department of Public Health Sciences, University of North Carolina at Charlotte, Charlotte, North Carolina.

## Abstract

**Introduction:**

In 2003, Barbados, a developing country with universal health care, launched the Barbados Strategic Plan for Health, a national intervention to promote public health. Teachers, health educators, and clinicians worked to improve children’s health, with particular focus on asthma and diabetes. We studied this intervention by using data on preventable hospitalization, an indicator that assesses both the overall effectiveness of public health and access to primary health care. The purpose of this study was to assess the Barbados Strategic Plan for Health by measuring rates of preventable hospitalization among children. Few researchers have studied these hospitalizations for children, and only 1 study has done so in a developing country.

**Methods:**

We calculated annual (2003–2008) population-based rates of preventable hospitalizations from birth through age 19, both summary and disease-specific, for the 5 conditions that define the indicator for children: asthma, diabetes, gastroenteritis, urinary tract infection, and perforated appendix.

**Results:**

Across the 6 years, the population rates of preventable hospitalizations increased 115.4% for boys and 67.2% for girls (both *P* < .001). Asthma accounted for much of the increase. Regression analysis indicated that the average annual increase in asthma hospitalization for boys was 0.45 per 1,000, an average annual increase of 20.6% of the baseline rate. These results suggest generally increasing rates of hospitalization for asthma for boys. There was no evidence of a corresponding rate trend for girls.

**Conclusion:**

Results suggest an opportunity to improve public health education and access to primary health care. Public health professionals in developing countries can use the approaches of this study to evaluate initiatives to improve child health.

## Introduction

In 2003, Barbados launched the Barbados Strategic Plan for Health, a national intervention to control disease and promote public health. Teachers, public health educators, and clinicians worked to improve public health, particularly for chronic diseases such as asthma and diabetes (1). We assessed this intervention by measuring preventable hospitalization, also called hospitalization for ambulatory care-sensitive conditions (2–10). Few researchers have studied these hospitalizations for children (4–9), and only 1 study has examined these hospitalizations in a developing country (5).

The preventable hospitalization indicator measures both public health and access to primary health care. Primary health care is a major contributor to childhood public health. For example, the Centers for Disease Control and Prevention (CDC) recommends a series of age-specific early and periodic screening, diagnosis, and treatment (EPSDT) visits for children insured by Medicaid, consisting of immunizations, blood lead evaluations, nutritional evaluation, parental guidance, and other preventive services (11). Public health agencies, including the CDC, increasingly collaborate with the medical care system to promote public health for children (11). The CDC selected preventable hospitalization as an area of focus of its 2011 and 2013 Health Disparities and Inequalities Reports (10), illustrating the usefulness of the indicator for public health. Although developing countries such as Barbados have limited systems for collecting and analyzing health data, and for making such data available to researchers, we demonstrate how public health officials can use the preventable hospitalization indicator to assess public health in a developing country.

Barbados, the easternmost Caribbean island, has a population of 289,700 (12). The infant mortality rate is 10.9 per 1,000 live births; life expectancy, at 75 years, is among the highest of the Caribbean islands (12). About 19% of the population is below age 15 (12). Nearly 93% of the population is of African descent. The literacy rate, defined as people aged 15 years or older who ever attended school, is nearly 100% (12). The epidemiological profile of Barbados is similar to those of developed countries (2,13). Chronic diseases have replaced nutritional deficiencies and infectious and parasitic diseases as the major health challenge (2,13). Details of the health care system are available (2,13). Barbados provides universal health care for all residents, covering most costs of outpatient care, hospitalization, and other services (2,13,14). Eight polyclinics and 4 satellite clinics provide free primary care, health education, and nutrition counseling to about 110,000 residents annually (2,13,14). Medications in the National Drug Formulary are free at all public health care facilities and in private clinics to Barbados residents age 16 or younger (2,14).

Asthma is one of the conditions that define preventable hospitalization for children. Barbados has one of the highest rates of asthma in the world (15). About 20% of children ages 6 and 7 have asthma (16), compared with about 14% in the United States (17). Some researchers believe that airborne African dust contributes to asthma in Caribbean countries (15). The hospitalization of children for diabetes is also often considered preventable. Childhood obesity and diabetes are increasing in Barbados (18).

On the basis of the World Health Organization’s definition of health as a basic human right (13), Barbados developed the Barbados Strategic Plan for Health in 2002 to address chronic diseases and rising health care costs and began implementing the plan in 2003 (1,2). Goals for children were to improve health and quality of life, nutrition and physical status, and air quality and to better manage health in outpatient settings to limit hospitalizations (1). Beginning in 2003 the Ministry of Health trained teachers, nurses, and physicians in an effort to educate all children and parents about asthma and diabetes (1). There is evidence that government officials and physicians designed the strategic plan with limited community involvement, and the plan focused principally on disease management rather than comprehensive health promotion (13). Nonetheless, the plan represented a substantial national effort to improve children’s health (1).

## Methods

In Barbados, as in many developing countries, researchers have limited access to health data. We obtained data through a special arrangement with the Barbados Ministry of Health. We used data representing all hospitalizations for children from birth through age 19 from 2003 through 2008 (n = 5,975).

Effective public health efforts and timely access to primary health care should limit preventable hospitalizations (2–11). We studied 5 pediatric ambulatory care-sensitive conditions: asthma, short-term complications of type 1 and type 2 diabetes, gastroenteritis, urinary tract infection, and perforated appendix (3). Consistent with most related studies, we combined hospitalizations for all 5 ambulatory care-sensitive conditions to examine total rates (6–9). We also examined the rate for each condition (2,6).

We identified preventable hospitalizations by using the pediatric ambulatory care-sensitive conditions definitions of the US Agency for Healthcare Research and Quality (AHRQ) (3,19). The definitions use *International Classification of Diseases, Ninth Revision, Clinical Modification* (ICD-9-CM) codes. Barbados uses *International Classification of Diseases, Tenth Revision, Clinical Modification* (ICD-10-CM) codes. We mapped the ICD-9-CM codes to ICD-10-CM codes by using a procedure developed by the US Centers for Medicare and Medicaid Services (20).

The data included narrative notes with diagnosis information. We used the narratives in addition to the diagnosis codes because the record of diagnoses in Barbados may be incomplete, as in many developing countries, if only the ICD-10-CM diagnosis codes are considered. We scanned the narratives electronically by using software we developed to identify preventable hospitalizations. Three authors (J.H.B-E., J.N.L., S.B.L.), with a combined 37 years of experience as health services researchers, participated in designing the software. The algorithm that identified these hospitalizations scanned all text for key words associated with preventable hospitalization diagnoses, such as asthma, and for abbreviated versions and variant spellings of such key words. Extensive testing on actual narratives with known diagnoses indicated that the electronic scans captured data on all relevant diagnoses. We validated each hospitalization that the electronic scan identified as preventable by reading the text of the relevant narrative. The narrative notes identified about 17.5% of preventable hospitalizations. Including those records in the analysis reduced the risk that the results might be biased by missing information in the ICD-10-CM diagnosis record.

We studied 74,041 children by using population estimates from the Barbados Census: 49% girls and 51% boys. We estimated mid-year population sizes for girls and boys for each year from 2003 through 2008. First, we obtained unpublished mid-year estimates from the Barbados Statistical Services for each year. These data estimated the size of the population of children from birth through age 19 but did not include separate estimates for girls and boys. Next, we identified the proportions of girls and boys in the 1980, 1990, and 2000 Barbados Census. Proportions of girls and boys did not vary meaningfully among these measures; therefore, it was reasonable to apply those proportions to the mid-year population estimates to obtain the number of girls and boys for each year.

We defined hospitalization rates separately for girls and boys as the ratio of the number of preventable hospitalizations to each 1,000 children in the population. We calculated preventable hospitalization rates on the basis of 504 of such hospitalizations for girls and 842 for boys. Because the rates are based on counts of hospitalizations, we used Poisson regression to estimate relative rates and 95% confidence intervals. We compared the results for 2004 through 2008 to results for 2003. We used α level *P* < .05 to identify significant results. We used SAS 9.2 (SAS Institute, Inc) for all analyses. Our study was approved by the institutional review board of the University of North Carolina at Charlotte and the Ethics Committee at Queen Elizabeth Hospital in Barbados, which accounts for almost all hospitalizations in Barbados.

## Results

### Preventable hospitalization across the study period

 The rate of preventable hospitalization for girls in 2003 was 1.95 per 1,000 (Table 1). The rate for girls did not differ significantly from 2003 in 2004 and 2007. The rate was modestly lower in 2005 than in 2003 and modestly higher in 2006. The rate was significantly higher in 2008, 3.26 (RR, 1.67; 95% CI, 1.25–2.24). For boys in 2003 the rate was 2.79 per 1,000. In 2004, the rate did not differ significantly from 2003; it was lower in 2005 (1.75 [RR, 0.62; 95% CI, 0.46–0.85]) and significantly higher in 2006 (4.65 [RR, 1.66; 95% CI, 1.31–2.12]), 2007 (4.13 [RR, 1.48; 95% CI, 1.15–1.89]), and 2008 (6.01 [RR, 2.15; 95% CI, 1.71–2.71]). Thus, for boys, preventable hospitalization generally increased across the study years, a 115.4% increase, from 2.79 to 6.01 (*P* < .001). The corresponding increase for girls was 67.2%, from 1.95 to 3.26 (*P* < .001). Preventable hospitalization rates were 19.1% of all hospitalizations for girls and 25.3% of all hospitalizations for boys.

**Table 1:** Risk of Hospitalization for Ambulatory Care-Sensitive Conditions for Girls and Boys, Birth Through Age 19, Barbados, 2003–2008, By Year^a^

Year	Girls			Boys		
	Rate per 1,000	Relative Risk (95% Confidence Interval)	*P* Value	Rate per 1,000	Relative Risk (95% Confidence Interval)	*P* Value^b^
2003	1.95	1 [Reference]		2.79	1 [Reference]	Reference
2004	2.11	1.08 (0.78–1.49)	.63	2.99	1.03 (0.79–1.35)	.81
2005	1.37	0.70 (0.49–1.01)	.06	1.75	0.62 (0.46–0.85)	.003
2006	2.62	1.34 (0.99–1.83)	.06	4.65	1.66 (1.31–2.12)	<.001
2007	2.45	1.26 (0.92­–1.72)	.15	4.13	1.48 (1.15–1.89)	.002
2008	3.26	1.67 (1.25–2.24)	<.001	6.01	2.15 (1.71–2.71)	<.001

a Data source is Queen Elizabeth Hospital, Barbados, 2003–2008, and Barbados Census 2000.

b *P* value is for comparison of the rate for the given year to the rate for 2003.

### Preventable hospitalization for the 5 ambulatory care-sensitive conditions

Compared with 2003, asthma hospitalization rates were lower for girls in 2005 (RR, 0.43; 95% CI, 0.26–0.71) and higher in 2008 (RR, 1.67; 95% CI, 1.18–2.36) (Table 2). Girls also had higher rates of perforated appendix in 2005, 2006, and 2007 relative to 2003. In addition, girls had higher rates of gastroenteritis in 2006 relative to 2003 (RR, 2.87; 95% CI, 1.35–6.13). For boys, compared with 2003, asthma rates were lower in 2005 (RR, 0.56; 95% CI, 0.39–0.80), and significantly higher in 2006 (RR, 1.46; 95% CI, 1.11–1.94) and 2008 (RR, 2.23; 95% CI, 1.72–2.90) (Table 3). Relative to 2003, boys also had higher rates for gastroenteritis in 2006 (RR, 3.64; 95% CI, 1.74–7.60), 2007 (RR, 5.38; 95% CI, 2.64–10.9), and 2008 (RR, 2.30; 95% CI, 1.05–5.02) (Table 3).

**Table 2:** Risk of Hospitalization for 5 Ambulatory Care-Sensitive Conditions, Girls, Birth Through Age 19, 2003–2008^a^

Condition and Year	Rate per 1,000 Hospitalizations	Relative Risk (95% Confidence Interval)	*P* Value^b^
**Asthma**			
2003	1.40	1 [Reference]	Reference
2004	1.26	0.90 (0.6–1.34)	.61
2005	0.60	0.43 (0.26– 0.71)	.001
2006	1.12	0.80 (0.53–1.21)	.29
2007	0.95	0.68 (0.44–1.05)	.08
2008	2.34	1.67 (1.18–2.36)	.004
**Diabetes**			
2003	0.11	1 [Reference]	Reference
2004	0.05	0.50 (0.09–2.72)	.42
2005	0.08	0.75 (0.17–3.34)	.70
2006	0.05	0.50 (0.09–2.72)	.42
2007	0.22	1.99 (0.60–6.59)	.26
2008	0.08	0.74 (0.17–3.32)	.70
**Gastroenteritis**			
2003	0.25	1 [Reference]	Reference
2004	0.19	0.78 (0.29–2.08)	.62
2005	0.19	0.77 (0.29–2.08)	.61
2006	0.71	2.87 (1.35–6.13)	.006
2007	0.60	2.43 (1.12–5.27)	.03
2008	0.41	1.65 (0.72–3.77)	.24
**Perforated appendix**			
2003	0.14	1 [Reference]	Reference
2004	0.38	2.79 (1.0­1–7.76)	.05
2005	0.46	3.39 (1.25–9.18)	.02
2006	0.49	3.58 (1.33–9.64)	.01
2007	0.57	4.17 (1.57–11.1)	.004
2008	0.35	2.58 (0.92–7.22)	.07
**Urinary tract infection**			
2003	0.05	1 [Reference]	Reference
2004	0.25	4.49 (0.97–20.80)	.06
2005	0.03	0.50 (0.05–5.49)	.57
2006	0.25	4.48 (0.97–20.70)	.06
2007	0.11	1.99 (0.36–10.80)	.43
2008	0.08	1.49 (0.25–8.89)	.67

a Data source: Queen Elizabeth Hospital, Barbados, 2003–2008; Barbados Census 2000.

b *P* value is for comparison of the rate for the given year to the rate for 2003.

**Table 3:** Risk of Hospitalization for 5 Ambulatory Care-Sensitive Conditions, Boys, Birth Through Age 19, 2003–2008^a^

Condition and Year	Rate per 1,000 Hospitalizations	Relative Risk (95% Confidence Interval)	*P* Value^b^
**Asthma**			
2003	2.18	1 [Reference]	Reference
2004	2.15	0.98 (0.72–1.34)	.91
2005	1.22	0.56 (0.39–0.80)	.002
2006	3.20	1.46 (1.11–1.94)	.008
2007	2.24	1.02 (0.76–1.39)	.88
2008	4.88	2.23 (1.72–2.90)	<.001
**Diabetes**			
2003	0.11	1 [Reference]	Reference
2004	0	NA	<.001
2005	0.05	0.50 (0.09–2.71)	.42
2006	0	NA	<.001
2007	0.03	0.25 (0.03–2.21)	.21
2008	0.05	0.49 (0.09–2.69)	.41
**Gastroenteritis**			
2003	0.24	1 [Reference]	Reference
2004	0.48	1.99 (0.89–4.43)	.09
2005	0.21	0.88 (0.34–2.29)	.80
2006	0.87	3.64 (1.74–7.60)	.001
2007	1.29	5.38 (2.64–10.9)	<.001
2008	0.55	2.30 (1.05–5.02)	.04
**Perforated appendix**			
2003	0.21	1 [Reference]	Reference
2004	0.19	0.87 (0.32–2.4)	.80
2005	0.21	0.99 (0.37–2.65)	.99
2006	0.48	2.23 (0.97–5.13)	.06
2007	0.47	2.22 (0.97–5.11)	.06
2008	0.31	1.48 (0.60–3.61)	.40
**Urinary tract infection**			
2003	0.05	1 [Reference]	Reference
2004	0.08	1.49 (0.25–8.94)	.66
2005	0.08	1.49 (0.25–8.92)	.66
2006	0.11	1.98 (0.36–10.80)	.43
2007	0.11	1.98 (0.36–10.80)	.43
2008	0.21	3.94 (0.84–18.50)	.08

a Data source: Queen Elizabeth Hospital, Barbados, 2003–2008, and Barbados Census 2000.

b *P* value is for comparison of the rate for the given year to the rate for 2003.

Regression analysis, shown for asthma for boys in the Figure, indicated that the average annual increase in asthma hospitalization for boys was 0.45/1,000 (*R* ^2^ = 0.45), an average annual increase of 20.6% of the baseline rate. Thus, rates of hospitalization for asthma for boys generally increased. The corresponding result for girls also suggested increasing rates, an average annual increase of 0.12/1,000 (*R* ^2 ^= 0.15); however, that result was influenced by the high 2008 rate, without which there was no evidence of a rate trend.

**Figure:**
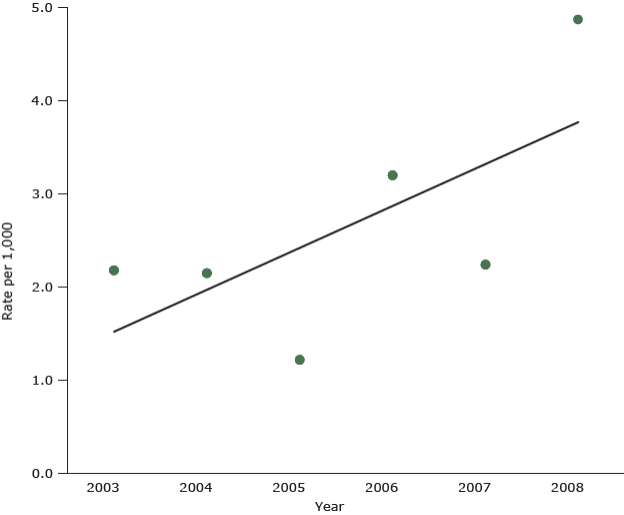
Potentially preventable hospitalization for asthma, Barbados, 2003–2008, males aged birth to 19 years. The diagonal line shows the results of an ordinary least squares regression analysis of potentially preventable hospitalization for asthma, Barbados, 2003–2008, for males birth to 19 years. Data source: Queen Elizabeth Hospital, Barbados, 2003–2008; Barbados Census 2000. **Table:** 

Year	Rate per 1,000 Hospitalizations
2003	2.18
2004	2.15
2005	1.22
2006	3.20
2007	2.24
2008	4.88

## Discussion

Our study contributes relevant information for assessing a national public health intervention in Barbados. We studied one aspect of the intervention, preventable hospitalization for children; it was not our purpose to study the effectiveness of specific characteristics of the intervention. Doing so was beyond the scope of this study. Rather, our goal was to illustrate how the preventable hospitalization indicator could be used to better understand public health in a developing country.

Overall rates of preventable hospitalization for children increased substantially during the 6 years of our study despite a universal health care system and an intervention to promote children’s health (1,13). A large proportion of the increase was due to asthma and gastroenteritis, particularly for boys. Asthma is one of the most prevalent ambulatory care-sensitive conditions (4–6). The higher asthma hospitalization rate for boys than for girls is consistent with previous research in Barbados and may be due to greater exposure to outdoor allergens for boys (16). The results suggest an opportunity to improve access to primary health care for children in Barbados, particularly for boys. However, the national program to educate people about managing chronic diseases may have prompted more people to seek care. More care-seeking could increase the number of children diagnosed with ambulatory care-sensitive conditions and may have increased the number of hospitalizations for those conditions. Therefore, the increased rates of preventable hospitalization might indicate that children who required hospitalization were more likely to obtain that level of care after implementation of the Barbados Strategic Plan for Health. 

This study had limitations. The study was a descriptive analysis. Unmeasured factors such as rural residence or the supply of health care professionals may have affected results. However, we have no reason to believe that the population distribution in rural areas or the supply of health care providers changed meaningfully during the study years. Aside from the Barbados Strategic Plan for Health, which was implemented beginning in 2003, there were no major policy or practice changes during the study period that would have been likely to affect the results (2,13).

We used a conservative definition of pediatric preventable conditions developed by the AHRQ (3,6,9). Other definitions have been used (4). However, at least 90% of the hospitalizations identified as potentially preventable by alternative definitions are typically included in the AHRQ indicator. Alternative definitions often include conditions such as hospitalization for dental conditions that appear so rarely that they do not alter results meaningfully. That the epidemiological profile of Barbados is similar to that of developed countries also supports our use of the AHRQ definition.

Researchers using the preventable hospitalization indicator in developed countries typically identify preventable hospitalizations using hospital discharge data. In Barbados such data are not reliably available. We used data on admission diagnoses. One strength of using admission data from a country with universal health care, one in which hospital reimbursements are not tied to discharge diagnosis coding, is that hospitals in such a system have no incentive to select or order diagnosis codes in ways designed to maximize reimbursements.

The data available for this study represented a period that began in 2003, when Barbados initiated its strategic plan. It would have been useful to include data from before that time. The addition of data from several years before the intervention would have permitted us to identify the trend in preventable hospitalization before the intervention and then to compare that trend with the results of the intervention. However, most public health initiatives require time to have measurable impact. The Barbados Strategic Plan for Health required the development of details and procedures for educating physicians, teachers, and health educators about the new national goals. It is unlikely that public health efforts, primary care practices, hospitalization protocols, and population behaviors changed substantially in the first year of the intervention. Thus, although it would be desirable to include data from before the intervention in the analysis, it was reasonable to begin the study with the first year of the intervention.

A study of primary care in Barbados conducted in 2007 concluded that the country had “excessive specialization” and a focus on disease programs that tend to serve the interests of physicians, as distinguished from a holistic approach to health based on community involvement (13). That study also found that the health care system concentrated resources on tertiary care in preference to prevention and health promotion (13). If that assessment is accurate, it would not be surprising if the results of the strategic plan evolved slowly, possibly with limited impact. Analysis of the organizational characteristics of the strategic plan development and implementation was beyond the scope of our study, and such an analysis would require different data and methods. It would be useful for researchers to assess whether a lack of community stakeholder involvement in the strategic plan development and implementation may have contributed to the results.

Another consideration is that changes in climate conditions, rainfall, or aeroallergens may have been responsible for the decrease in asthma hospitalizations in 2005; however, these factors in asthma attacks need further study (15,16). There were no major weather events or other environmental or social factors or meaningful changes in hospital bed capacity that could have influenced the variability in hospitalization rates. The proportion of pediatric hospitalizations that were potentially preventable did not differ greatly among the study years with the exception of 2005, when there were fewer asthma hospitalizations. Thus, the generally increasing rates of preventable hospitalization across the study years were part of a pattern of generally increasing hospitalization rates. Finally, as with most related studies (2,4–9), it took several years to obtain and analyze the data. This period was longer for the present study because Barbados, like many developing countries, has limited procedures for managing and releasing electronic health records and limited resources to respond to researchers’ data requests. It would be useful to replicate this analysis with more recent data.

In conclusion, results of this study suggest a substantial opportunity to improve asthma education for children and their parents in Barbados, particularly for boys. Barbados is a small country; however, the lessons learned from this study may be broadly applicable. For example, China faces an epidemic of obesity and diabetes along with respiratory conditions associated with air pollution. India’s growing middle class also has high rates of obesity, diabetes, and heart disease. Such countries can use preventable hospitalization to monitor both public health and access to medical care. As public health officials and clinicians in the United States increasingly recognize, both public health and medical care work better when they combine agendas and resources to prevent and better manage chronic diseases.

## References

[1] Barbados Ministry of Health. Health of a nation is the wealth of a nation: Barbados strategic plan for health 2002–2012. Bridgetown (BB):: Barbados Ministry of Health; 2003. http://apps.who.int/medicinedocs/en/d/Js18831en/. Accessed February 20, 2015.

[2] Bushelle-Edghill JH , Laditka JN , Laditka SB , Brunner Huber LR . Evaluating access to primary health care among older women and men in Barbados using preventable hospitalization. J Women Aging 2015;27(4):273–89. 10.1080/08952841.2014.950135 25651165

[3] Agency for Healthcare Research and Quality. Pediatric quality indicators overview. http://www.qualityindicators.ahrq.gov/Modules/pdi_overview.aspx. Accessed February 20, 2015.

[4] Flores G , Abreu M , Chaisson CE , Sun D . Keeping children out of hospitals: parents’ and physicians’ perspectives on how pediatric hospitalizations for ambulatory care-sensitive conditions can be avoided. Pediatrics 2003;112(5):1021–30. 10.1542/peds.112.5.1021 14595041

[5] Niti M , Ng TP . Avoidable hospitalisation rates in Singapore, 1991–1998: assessing trends and inequities of quality in primary care. J Epidemiol Community Health 2003;57(1):17–22. 10.1136/jech.57.1.17 12490643 PMC1732279

[6] Garg A , Probst JC , Sease T , Samuels ME . Potentially preventable care: ambulatory care-sensitive pediatric hospitalizations in South Carolina in 1998. South Med J 2003;96(9):850–8. 10.1097/01.SMJ.0000083853.30256.0A 14513978

[7] Laditka JN , Laditka SB , Probst JC . More may be better: evidence of a negative relationship between physician supply and hospitalization for ambulatory care sensitive conditions. Health Serv Res 2005;40(4):1148–66. 10.1111/j.1475-6773.2005.00403.x 16033497 PMC1361189

[8] Laditka SB , Johnston JM . Preventable hospitalization and avoidable maternity outcomes: implications for access to health services for Medicaid recipients. J Health Soc Policy 1999;11(2):41–56. 10.1300/J045v11n02_04 10620859

[9] Laditka JN , Laditka SB , Probst JC . Health care access in rural areas: evidence that hospitalization for ambulatory care-sensitive conditions in the United States may increase with the level of rurality. Health Place 2009;15(3):731–40. 10.1016/j.healthplace.2008.12.007 19211295

[10] Moy E , Chang E , Barrett M ; Centers for Disease Control and Prevention (CDC). Potentially preventable hospitalizations — United States, 2001–2009. MMWR Surveill Summ 2013;62(Suppl 3):139–43. 24264504

[11] Pittard WB 3d , Laditka JN , Laditka SB . Associations between maternal age and infant health outcomes among Medicaid-insured infants in South Carolina: mediating effects of socioeconomic factors. Pediatrics 2008;122(1):e100–6. 10.1542/peds.2007-1314 18595955

[12] Index Mundi. Barbados demographic profile 2014. http://www.indexmundi.com/barbados/demographics_profile.html. Accessed May 2, 2015.

[13] Rodney P , Copeland E . Safeguarding primary health care: a case study of Barbados. Soc Med 2009;4:204–9. http://www.socialmedicine.info/index.php/socialmedicine/article/view/336. Accessed June 19, 2015.

[14] Pan American Health Organization. Health systems profile: Barbados; 2008. Washington (DC): Pan American Health Organization. http://new.paho.org/hq/dmdocuments/2010/Health-System-Profile-Barbados-2008.pdf. Accessed July 11, 2015.

[15] Prospero JM , Blades E , Naidu R , Mathison G , Thani H , Lavoie MC . Relationship between African dust carried in the Atlantic trade winds and surges in pediatric asthma attendances in the Caribbean. Int J Biometeorol 2008;52(8):823–32. 10.1007/s00484-008-0176-1 18773225

[16] Depradine CA , Lovell EH . The incidence of asthmatic attacks in Barbados. West Indian Med J 2007;56(5):427–32. 18303755

[17] Bloom B , Jones LI , Freeman G . Summary health statistics for US children: National Health Interview Survey, 2012. Vital Health Stats 10 2013;(258)1–81. 24784481

[18] Yisahak SF , Beagley J , Hambleton IR , Narayan KMV ; IDF Diabetes Atlas. Diabetes in North America and the Caribbean: an update. Diabetes Res Clin Pract 2014;103(2):223–30. 10.1016/j.diabres.2013.11.009 24321468

[19] McDonald KM , Davies SM , Haberland CA , Geppert JJ , Ku A , Romano PS . Preliminary assessment of pediatric health care quality and patient safety in the United States using readily available administrative data. Pediatrics 2008;122(2):e416–25. 10.1542/peds.2007-2477 18676529

[20] Centers for Medicaid and Medicare Services. Road to 10: the small physician practice’s route to ICD-10; 2015. http://www.roadto10.org/. Accessed February 20, 2015.

